# Differences in Surgical Management of Corneal Perforations, Measured over Six Years

**DOI:** 10.1155/2017/1582532

**Published:** 2017-02-23

**Authors:** Katarzyna Krysik, Dariusz Dobrowolski, Anita Lyssek-Boron, Judyta Jankowska-Szmul, Edward A. Wylegala

**Affiliations:** ^1^Department of Ophthalmology with Pediatric Unit, St. Barbara Hospital, Trauma Center, Medykow Square 1, 41200 Sosnowiec, Poland; ^2^Clinical Departament of Ophthalmology, School of Medicine with the Division of Dentistry in Zabrze, Medical University of Silesia in Katowice, District Railway Hospital, Panewnicka 65 St., 40760 Katowice, Poland

## Abstract

*Purpose*. To report the surgical approach, anatomical and functional results, and complications in the group of patients with corneal perforation. *Materials and Methods*. 247 eyes with corneal perforation were operated on between January 2010 and July 2016. The three surgical procedures, dependent on size and location of perforation, were performed: full-sized penetrating keratoplasty, corneoscleral patch graft, and anterior lamellar keratoplasty. The eyes underwent the minimum 6-month follow-up visit. *Results*. Between January 2010 and July 2016, 247 surgeries were performed: 116 penetrating keratoplasties, 117 corneoscleral patch grafts, and 14 anterior lamellar keratoplasties. More than one procedure was necessary in 32 eyes. Final improvement of the visual acuity, within a gain of 2 or more lines with the Snellen test, was achieved in 56 operated eyes. To achieve better final visual acuity, 75 eyes required successive surgical treatment. Complications of the surgery comprised persistent epithelial defect, glaucoma or ocular hypertension, corneal oedema, graft melting, loose corneal sutures, reinfection, anterior synechiae and fibrinoid membranes, and endophthalmitis. In 26 eyes, the treatment failure was reported. *Conclusions*. There is no one general-purpose surgical technique to treat corneal perforations. The complex nature of this pathology remains the individual, careful but also very distinct and multifactorial approach.

## 1. Introduction

Corneal perforation is a common condition, and it can lead to profound vision loss and severe ocular morbidity. It can be caused by infection, bacterial and noninfectious ocular surface disorders, immune disorders, and trauma. Other conditions such as xerosis, exposure, neurotrophic disorders, corneal degeneration, and ectasia and surgical and toxic/keratolytic treatment can lead to corneal perforation. Primary or secondary epithelial defects facilitate the development of corneal inflammation with subsequent perforation [1, 2].

A diagnosis of corneal perforation does not always require immediate surgical treatment. The primary goal in repairing the corneal perforation is to achieve a watertight globe with structural integrity in order to prevent severe complications such as globe tissue prolapse, endophthalmitis, and glaucoma [1, 3]. Frequently, the surgical procedure first provides temporary treatment, and the final treatment to restore visual function involves a multistage surgical approach. The therapeutic procedure and its timing depend on various conditions, such as cause, location, size of perforation, corneal infiltration and melting, and state of internal globe tissues [1, 4–7].

The nonsurgical approach includes limitation of inflammation, the treatment of coexistent infection, the use of anticollagenase and antiglaucomatous treatment, and the optimization of epithelial healing (bandage soft contact lenses, autologous serum eye drops, and punctal occlusion) [1, 8]. Surgical treatment includes tissue adhesives, amniotic membrane transplants, conjunctival flaps, pericardial membranes, tarsorrhaphy, and therapeutic ptosis with botulinum toxin [1, 6, 9–12].

The surgical procedure of tectonic keratoplasty is used to restore globe integrity. This procedure includes full-thickness penetrating keratoplasty, anterior lamellar keratoplasty (ALK), and corneal patch grafts. Penetrating keratoplasty (PK) is limited to large perforations with extensive necrosis [2–5, 13, 14]. In peripheral corneal perforations and descemetoceles and when the perforation is relatively small (i.e., it does not require full-sized penetrating keratoplasty but is too large for tissue adhesive) [1, 15, 16], tectonic corneoscleral patch grafting is performed as a temporary or definitive treatment. Lamellar keratoplasty is performed in the case of leaking descemetocele, to reinforce the thinned and necrotic stroma, and in corneal perforations. The advantage of this method is lower risk of corneal rejection [17, 18].

The aim of this study is to report on a sample of patients who underwent surgical treatment for corneal perforation. We report on the surgical techniques, anatomical and functional results, and complications of treatment in this group of patients.

## 2. Materials and Methods

**Figure 1:**
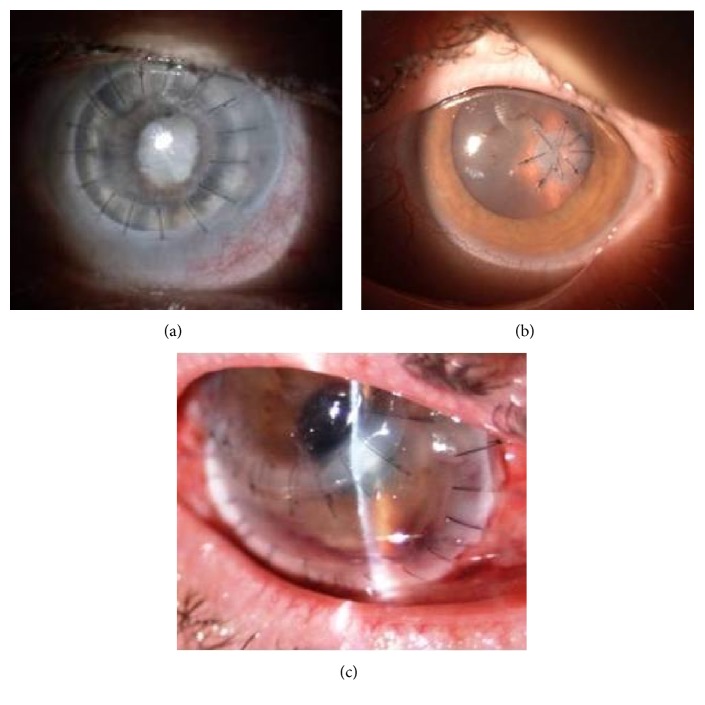
Surgical procedures applied in corneal perforations: (a) full-sized penetrating keratoplasty in fungal keratitis, (b) corneoscleral patch graft in RA patient, and (c) anterior lamellar keratoplasty in necrotizing peripheral keratitis.

This study was a retrospective review of the surgical treatment of 247 eyes with corneal perforation, which were operated between January 1, 2010, and July 31, 2016, at the Ophthalmology Department of Saint Barbara Hospital, Trauma Center, Sosnowiec, Poland. The analysed data from the medical records included demographics, medical history, corrected-distance preoperative and postoperative Snellen visual acuity, details, outcome and complications of surgery, and ocular integrity. All patients with diagnosed corneal perforation who underwent complete ocular examination were treated using one of three surgical techniques: full-sized penetrating keratoplasty (Figure 1(a)), corneal or corneoscleral patch grafts of varying sizes and shapes (Figure 1(b)), and anterior lamellar keratoplasty (Figure 1(c)). The main purpose of the treatment was to achieve the structural integrity of the globe. The choice of surgical procedure depended on the size and location of the perforation, the state of the internal ocular tissues, and intraocular infection. Surgeries were done as quickly as possible after diagnosis of disease origin was established and sufficient topical and general treatment was administered.

Size was classified according to three groups: small (0.1–2.5 mm diameter), medium (2.5–5.0 mm diameter), and large (over 5.0 mm diameter). Location was categorised as central (involving visual axis, ≤5.0 mm diameter of the central cornea), paracentral (zone over 5.0 mm up to 8.0 mm), or peripheral (≥8.0 mm, also including the limbus). If the perforation was small or medium and the visual axis was free of pathology, corneoscleral patch grafting was usually performed. For large perforations with intensive corneal melting or thinning, necrosis, and internal bulbar tissue injury, penetrating keratoplasty was the preferred surgical approach. Fresh corneas were applied for each trephination. The graft diameter was 2.5–12.0 mm, with an oversize of 0.5–1.0 mm, and the donor-recipient junction was sewn by 10-0 nylon interrupted sutures. Anterior lamellar therapeutic keratoplasties were performed for leaking descemetoceles, and they depended on the depth and severity of the corneal pathology. All surgical procedures were performed under local or general anaesthesia. The donor corneas originated from our own or cooperative tissue banks. For PK, we used a Hanna vacuum trephine system (Moria Inc., Antony, France); for ALK, a manual or automated technique was employed, using a femtosecond laser (FL-assisted lamellar keratoplasties) or microkeratome dissection (Hanna trephine). The freehand technique was applied for corneoscleral patch grafts. Additional treatment was given according to the aetiology. All patients were hospitalised for the first 3–7 days after operation and were followed up every two weeks for a period of two months, monthly for a minimum of six months, and at differing intervals thereafter.

## 3. Results

Between January 1, 2010, and July 31, 2016, 247 surgeries for corneal perforations were performed. This involved 125 procedures in the group of 81 females, where the mean age was 65.22 ± 15.4 (range 9–90 years), and 122 procedures in the group of males, where the mean age was 57.56 ± 17.07 (range 20–88 years). To restore ocular integrity, more than one procedure in each eye was required for 21 females (26%) and for 11 males (11%). Two patients presented bilateral perforation. A 69-year-old female with rheumatoid arthritis was operated four times, twice in both eyes with penetrating keratoplasty. Because of the size and location of the corneal perforation, penetrating keratoplasty was the method of choice. A 38-year-old male with primary immunodeficiency also presented bilateral perforation. For his right eye, two corneoscleral patch grafts and one penetrating keratoplasty were performed, and one penetrating keratoplasty and one anterior lamellar keratoplasty were performed for his left eye.

There were 125 surgical tectonic procedures in the female group with corneal perforation. This comprised 60 penetrating keratoplasties (48%), 56 corneoscleral patch grafts (45%), and 9 anterior lamellar keratoplasties (7%), including 3 FL-assisted procedures. In the male group of patients, 122 tectonic procedures were performed, comprising 56 penetrating keratoplasties (46%), 61 corneoscleral patch grafts (50%), and 5 anterior lamellar keratoplasties, including 2 FL-assisted procedures. The penetrating approach was chosen for cases with large, full-thickness stromal involvement (infections, melting). For cases with local lesions, we preferred patch grafts. Such eyes included peripheral perforations (29%) and central or paracental disorders (18.1%). Surgical host's bed preparation with removal of necrotic tissue was done in each case. Lamellar surgery was done; despite leakage for exposed Descemets' membrane, we were able to continue surgery with lamellar separation of the affected tissue.

Three main causes of corneal perforation were reported. The most common cause was infection (45%), mainly bacterial (76%); inflammatory conditions (38%), mainly autoimmune causes; and ocular trauma (17%), mainly alkali burns (86%). The indications and details of the surgical tectonic treatment of the corneal perforations are presented in Tables 1 and 2.

Initial treatment after surgery included broad-spectrum antibiotic/antimycotic (fluoroquinolones (moxiflixacin or levofloxacin), aminoglycosides (gentamycin), fluconazole, voriconazole, and amphotericin B); steroid (dexamethasone 5 times a day); lubricants (including autologous serum); and general antibiotics, steroids, immunosuppressive agents (azathioprine, mofetil mycophenolate). In majority of cases, the individual approach was confirmed by microbiologists and rheumatologists to establish balance between antimicrobial agents and immunosuppressive drugs. Modifications were applied according to progress of the disease. Topical antimicrobial therapy was routinely applied for 21 days, extended if needed. Steroid doses were lowered (each month 1 drop less) to stop before the 6th month of therapy. Instable epithelia needed persistent intensive lubrication up to each hour of administration.

Final improvement of visual acuity, within a gain of two or more lines with the Snellen test, was achieved in 56 operated eyes (22.7%), that is, for 29 females (52%) and 27 males (48%). VA improvement was observed in 8 of 14 in the lamellar keratoplasty group, 32 of 116 in the penetrating keratoplasty group, and 16 of 117 in the corneoscleral patch graft group. Initial VA ranged no light perception to 20/30 before treatment, and final VA ranged no light perception to 20/25.

Seventy-five eyes (30.4%) of 31 females and 44 males treated initially for corneal perforation required successive surgical treatment to achieve useful visual acuity. Table 3 presents the successive procedures performed to improve visual acuity.

There were various complications of the surgical treatment. The most common complication was persistent epithelial defect, reported in 121 eyes (49%). Many of them occurred in PK patients with a diameter of the graft greater than 5 mm (37.1%). There were patients with all types of infections (14.5%), autoimmune diseases (7.6%), and burns (7.6%). The main reason for persistent epithelium was decreased corneal sensitivity. The same reason with insufficient tearing was observed in the patch graft group suffering from autoimmune diseases. Both caused epithelium loss in patch graft performed for bacterial and viral ulcer (6.4%) or in disorders combined with rheumatoid diseases (8.0%). Treatment of erosions was based on application of 20% solution of autologous serum combined with an antibiotic agent and dexamethasone 3 times a day. In 17 eyes, additionally, an amniotic membrane was applied for 2 weeks to support reepithelialisation. Further evolution of persistent erosion to stromal melting caused a need of re-PK or PK in the PG/ALK group.

Graft melting was reported in 45 eyes (18.2%). This complication was common in PK and PG groups. 10 PKs for bacterial and one for fungal infection developed necrosis in the periphery of the graft. The treatment focused on intensive antimicrobial treatment. In failed cases, 8 re-PKs were done with a large oversize to cover infected stroma. Patch graft performed for perforations due to local infections developed melting in 16 cases; 12 patients with rheumatoid arthritis and 4 with neurotrophic ulcer also presented signs of graft necrosis with involvement of surrounding host's stroma in 12 eyes. This enlargement of necrosis caused a need of penetrating keratoplasty in 12 eyes. Corneal suture loosening was reported in 43 eyes (17.4%). This factor was considered an initial factor in melts at the periphery of small grafts if combined with autoimmune origin of the primary disease.

Glaucoma or ocular hypertension was reported in 69 eyes (28%). Majority of them were treated with 1 or 2 topical agents (timolol 0.5%, brimonidine) or referred to trabeculectomy in 16 cases.

Corneal oedema was reported in 56 eyes (22.7%): 12 cases of PK and 44 patch grafts. Persistent oedema in PK patients in 6 cases caused a need to exchange the lenticule. 18.6% of eyes developed stromal vasculature. Risk of that complication rises with the diameter of the graft and the distance to the limbus. Keratoplasties with a diameter over 8 mm developed neovascularisation in a graft-host interface in 14 cases for overall 32 such interventions. Patch grafts in autoimmune disorders (rheumatoid arthritis, Mooren's ulcer) presented vascular ingrowths in graft bed in over 50% of cases, commonly present in periphery.

Reinfections, as mentioned before, were reported in total 34 eyes (13.7%), anterior synechiae and fibrin retrocorneal membranes, reported in 17 eyes (6.8%), and endophthalmitis, reported in 7 eyes (3%). In 26 eyes (10.5%) with corneal perforation, despite repeated surgical treatment and intensive pharmacological treatment, treatment failure was reported. There was loss of light perception in 11 eyes (4.4%), persistent hypotony in 4 eyes (1.6%), and retinal detachment in 5 eyes (2.0%); in 4 eyes (1.6%), evisceration was necessary.

## 4. Discussion

Our study aimed to achieve and maintain anatomical and functional integrity of the ocular surface. This was possible using corneal donor tissues originating from ocular tissue banks. We used three types of tectonic corneal grafts: penetrating keratoplasty, anterior lamellar keratoplasty, and corneoscleral patch graft. The grafts varied in size, shape, and position, depending on the aetiology, extensity, location, and infiltration of the corneal tissue. In addition, the state of the internal bulbar tissues determined the time and method of the surgical approach. We operated on various corneal perforations that were the result of infection (mainly bacterial), inflammatory disease (mainly collagen vascular diseases), and trauma.

Surgical techniques such as lamellar keratoplasties, patch grafting, and keratolimbal allografting have become more popular in recent times [2, 19, 20]. In our department, corneoscleral patch grafts have been used in peripheral or less frequently central corneal perforations only since 2006. Since 2010, our department has also performed FL-assisted anterior lamellar keratoplasties for corneal perforations.

In the current study, we reported that the therapeutic approach to corneal perforation is challenging and unpredictable. The tectonic keratoplasties used in our study are often more favourable in cases of stomal involvement by necrosis than local or general medication, using adhesive tissues or other forms of treatment. Frequently, there is only one possible approach to restore ocular surface integrity. Removal of infected tissue decreases the risk of endophthalmoitis (the rate of that complication is really low—3%). In autoimmune diseases, therapeutic graft restores stromal integrity and delivers the basement membrane of the epithelium. However, it does not exclude support of epithelial healing, which in cases with necrosis is very slow.

A very important aspect of surgical treatment for corneal perforation is the availability of tissue necessary for the procedure. When there is no donor cornea, the amniotic membrane should be accesible or we need to use a conjunctival flap to restore ocular surface integrity temporarily or definitely [6, 9].

In our sample of patients with corneal perforation who were treated with lamellar keratoplasty, the advantage of this surgical technique conferred lower risk of subsequent allograft rejection and graft failure. This is the result of the preservation of recipient endothelium [5, 18, 21]. On the other hand, there is a risk of stromal or epithelial graft failure, with subsequent visual loss, in this type of corneal graft [21]. Our patients who underwent surgical treatment with ALK more frequently demonstrated an improvement in visual acuity than did patients after other treatment methods (57% versus 27.6% after penetrating keratoplasty and 13.7% after corneoscleral patch graft) [18]. The size, location, and aetiology of the corneal perforation also contribute to visual acuity [4, 6].

Penetrating keratoplasty also remains more popular than anterior lamellar keratoplasty because of the lower risk of donor tissue injuries, such as Descemet membrane perforation [18, 20].

In cases of corneal perforation originating from infectious keratitis, we strongly advise antibacterial, antifungal, or antiviral general and topical intensive medication as the first treatment before surgical treatment. This is not always possible, and the sequence of procedures and timing of the surgery also depend on the control and severity of the infection and globe integrity [1, 4].

The functional result of the surgical treatment of the corneal perforation is often secondary to anatomical disorders. Visual outcomes are not so important in the first surgical approach; a stable, watertight ocular surface is the first goal of treatment of corneal perforation. Grafts in eyes with perforations have usually active primary ocular disease. It is better and more predictive in results when the ocular surface is quiet. Active infection, inflammation, or other conditions present before remained immediately after surgery.

To achieve the best final results of treatment, we had to perform different and often multistage surgical procedures, spread over time [4]. Repeat penetrating keratoplasty was the most frequent treatment in our study group, because of corneal scars and allograft rejection, especially in eyes with active keratitis. In 14.7% of reoperated eyes, repeat penetrating keratoplasty was performed simultaneously with cataract extraction and PC IOL implantation. Cataract surgery alone or secondary intraocular lens implantation in aphakic eyes was also beneficial for restoring better visual acuity. With more complicated cases after tectonic keratoplasty, such as in 7 eyes (9.3%) with previous intrabulbar inflammation with inflammatory membranes and vitreoretinal tractions or retinal detachment, pars plana vitrectomy was necessary to improve visual outcome.

In summary, our results of the surgical treatment of corneal perforations show that there is no one-size-fits-all surgical approach to corneal perforation. Because of the complex nature of the tectonic indications for surgery and their modalities, it is very difficult to present final and unequivocal forms of treatment. The choice of surgical technique and the results of surgical treatment depend on numerous factors. The further development of surgical and lamellar surgical techniques applied in corneal perforations is also necessary.

## Figures and Tables

**Table 1:** Surgical techniques and indications for surgery in corneal perforation.

Characteristics	Total *n* (%) (*n* = 247)	Female *n* (%) (*n* = 125)	Male *n* (%) (*n* = 122)
*Penetrating * *keratoplasty (PK)*	116 (47.0)	60 (52)	56 (48)
Indication for surgery			
Infection	60 (24.3)	36 (28.8)	24 (19.7)
Inflammation	33 (13.4)	17 (13.6)	16 (13.1)
Ocular trauma	23 (9.3)	7 (5.6)	16 (13.1)
*Patch graft (PK)*	117 (47.3)	56 (48)	61 (52)
Indication for surgery			
Infection	47 (19.0)	22 (17.6)	25 (20.5)
Inflammation	54 (21.9)	27 (21.6)	27 (22.1)
Ocular trauma	16 (6.5)	7 (5.6)	9 (7.4)
*ALK*	14 (5.7)	9 (64)	5 (36)
Indication for surgery			
Infection	4 (1.6)	3 (2.4)	1 (0.82)
Inflammation	7 (2.8)	5 (4.0)	2 (1.64)
Ocular trauma	3 (1.2)	1 (0.8)	2 (1.64)

**Table 2:** Subgroups of indications for each surgical treatment.

Indications	Subgroup	PK *n* (%)	PG *n* (%)	ALK *n* (%)
Infections	Bacterial	45 (38.7)	41 (35.9)	2 (14.2)
	Fungal	12 (10.3)	6 (5.1)	2 (14.2)
	Viral	3 (2.5)	0	0

Inflammatory disorders	Rheumatoid	26 (22.4)	33 (28.2)	2 (14.2)
	Neurotrophic	5 (4.3)	10 (8.5)	3 (21.4)
	Mooren's ulcer	0	8 (6.8)	2 (14.2)
	Lyell's syndrome	1 (0.8)	2 (1.7)	0
	OCP	1 (0.8)	1 (9.8)	0

Traumatic	Alkali burn	12 (10.3)	7 (5.9)	3 (21.4)
	Melting	10 (8.6)	7 (5.9)	0
	Acidic burn	1 (0.8)	2 (1.7)	0

**Table 3:** Successive surgical treatment after primary tectonic keratoplasty.

Surgical technique	Total *n* (%) (*n* = 75)	Female *n* (%) (*n* = 31)	Male *n* (%) (*n* = 44)
PK	15 (20.0)	9 (29.0)	6 (13.6)
PK with cataract surgery and PC IOL implantation	11 (14.7)	4 (12.9)	7 (15.9)
Cataract surgery with PC IOL implantation	19 (25.3)	8 (25.8)	11 (25.0)
Secondary in-the-bag PC IOL implantation	5 (6.7)	4 (12.9)	1 (2.3)
Secondary in-the-sulcus PC IOL implantation	4 (5.3)	0	4 (9.1)
Transscleral fixation of IOL	8 (10.7)	2 (6.5)	6 (13.6)
Iridoplasty	6 (8.0)	2 (6.5)	4 (9.1)
Pars plana vitrectomy (PPV)	3 (4.0)	1 (3.2)	2 (4.6)
PPV with cataract surgery and PC IOL implantation	4 (5.3)	1 (3.2)	3 (6.8)
